# Effects of A 60 Hz Magnetic Field of Up to 50 milliTesla on Human Tremor and EEG: A Pilot Study

**DOI:** 10.3390/ijerph14121446

**Published:** 2017-11-24

**Authors:** Shirin Davarpanah Jazi, Julien Modolo, Cadence Baker, Sebastien Villard, Alexandre Legros

**Affiliations:** 1 Human Threshold Research Group, Imaging Program, Lawson Health Research Institute, London, ON N6A 4V2, Canada; sdavarpa@uwo.ca (S.D.J.); julien.modolo@inserm.fr (J.M.); cbaker55@uwo.ca (C.B.); svillard@uwo.ca (S.V.); 2 Department of Medical Biophysics, Western University, London, ON N6A 5C1, Canada; 3 LTSI U1099, INSERM, Université de Rennes, 35042 Rennes, France; 4 School of Kinesiology, Western University, London, ON N6A 5B9, Canada; 5 Department of Medical Imaging, Western University, London, ON N6A 5W9, Canada

**Keywords:** human, electroencephalography, extremely low frequency, magnetic field, physiological tremor

## Abstract

Humans are surrounded by sources of daily exposure to power-frequency (60 Hz in North America) magnetic fields (MFs). Such time-varying MFs induce electric fields and currents in living structures which possibly lead to biological effects. The present pilot study examined possible extremely low frequency (ELF) MF effects on human neuromotor control in general, and physiological postural tremor and electroencephalography (EEG) in particular. Since the EEG cortical mu-rhythm (8–12 Hz) from the primary motor cortex and physiological tremor are related, it was hypothesized that a 60 Hz MF exposure focused on this cortical region could acutely modulate human physiological tremor. Ten healthy volunteers (age: 23.8 ± 4 SD) were fitted with a MRI-compatible EEG cap while exposed to 11 MF conditions (60 Hz, 0 to 50 mT_rms_, 5 mT_rms_ increments). Simultaneously, physiological tremor (recorded from the contralateral index finger) and EEG (from associated motor and somatosensory brain regions) were measured. Results showed no significant main effect of MF exposure conditions on any of the analyzed physiological tremor characteristics. In terms of EEG, no significant effects of the MF were observed for C1, C3, C5 and CP1 electrodes. However, a significant main effect was found for CP3 and CP5 electrodes, both suggesting a decreased mu-rhythm spectral power with increasing MF flux density. This is however not confirmed by Bonferroni corrected pairwise comparisons. Considering both EEG and tremor findings, no effect of the MF exposure on human motor control was observed. However, MF exposure had a subtle effect on the mu-rhythm amplitude in the brain region involved in tactile perception. Current findings are to be considered with caution due to the small size of this pilot work, but they provide preliminary insights to international agencies establishing guidelines regarding electromagnetic field exposure with new experimental data acquired in humans exposed to high mT-range MFs.

## 1. Introduction

Alternating currents at 50 (in Europe) or 60 (in North America) Hz travel through power lines over long distances and are circulating in our domestic appliances. Thus, extremely low frequency (ELF, <300) magnetic fields (MFs) are produced around these power lines and gradually decrease as distance from the cables increases. Power line workers that operate in the vicinity of these lines and the general public who uses every-day electrical appliances, such as hair dryer or shavers, are exposed to different levels of MF that can sometimes reach 1000 to 2000 μT [1,2,3,4]. Over the past few decades, various physiological, neurophysiological and behavioral measures have been used to investigate the potential effects of ELF MFs. For example, Ghione et al. [5] investigated the effect of 90 min ELF MF (50 Hz, 0.04 or 0.08 mT) exposure on resting electroencephalography (EEG, brain electrical activity), pain perception, blood pressure and heart rate of healthy individuals. The authors demonstrated that, while blood pressure and heart rate did not show reliable changes, resting occipital alpha rhythm (8–12 Hz) increased as a result of MF exposure. The authors suggested that such increase was the result of higher neural activity level in the occipital region leading to increased inward attention. In addition, pain sensitivity increased which was indicative of a sensory gating process in the opioidergic system resulting from MF exposure. Other studies that used similar MF frequency with different intensity and short duration (<5 s) have replicated these results [6,7,8,9]. In addition to the brain’s neural activity (i.e., EEG profile), human motor behavior (i.e., standing balance, physiological tremor) may be modulated by long- (=15 min) and short-term (2 min or less) ELF MF exposure. More specifically, results showed a reduction in anteroposterior standing balance oscillation as well as an increase in physiological tremor amplitude because of a 50 and 60 Hz low mT-range (i.e., <1.8 mT) MF exposure [10,11,12]. Notably, the exposure level employed in the above-mentioned studies were kept at a low intensity mT-range. As such, previous studies that have examined human neurophysiology under high levels (8 mT or higher) of ELF MF exposure have only looked at magnetophosphene perception (flickering visual perceptions while the eyes are closed) [13,14,15]. It was reported that magnetophosphene perception is dependent on the frequency and flux density of the MF; as well as background lighting levels and adaptability to light/darkness.

A sensitive neuromotor outcome offering an original window on the potential neurophysiological impact of an ELF MF exposure applied to the human motor cortex is physiological tremor. Physiological tremor is present while at rest (resting tremor), moving (kinetic tremor) or when maintaining the position of an extremity against gravity (postural tremor). Physiological tremor is known to originate from three neurophysiological factors: direct contribution of the central nervous system associated with motor unit firing properties (8–12 Hz), negative feedback loops produced through stretch reflex mechanisms (10 Hz) and mechanical properties of the involved body segment [15,16]. Additionally, physiological tremor in the fingers is low in amplitude (less than 0.1 mm in displacement) and relatively high in frequency (7–12 Hz; up to 27 Hz when including mechanical factors) [16,17]. One way of evaluating physiological tremor is through measuring a specific type of tremor referred to as postural tremor. Postural tremor qualifies as tremor occurring in a segment while maintaining a fixed position against gravity. In our study, it specifically refers to tremor at the tip of the index finger while pointing ahead. Postural tremor can be increased by removing visual input. Human neurophysiology is highly sensitive to modifications in the proximal environment, which can affect postural tremor in particular. Factors such as caffeine intake, temperature, limb position, alcohol, mercury and MF exposure can influence postural tremor [11,18,19,20]. In an earlier study by our group, the effect of a 1 mT_rms_, 50 Hz MF exposure on postural tremor was reported [11]. More specifically, participants had to maintain their index finger in an extended position for durations of 62 s while tremor characteristics (i.e., amplitude and frequency) were measured. Results showed a significant time-dependent effect of MF exposure on tremor, namely a decrease in postural tremor amplitude over time. However, these findings demonstrated effects of ELF MF exposure at low levels and thus, the potential acute effects of high levels of ELF MF on postural tremor in humans remains unexplored.

Further, international agencies such as the International Commission on Non-Ionizing Radiation Protection (ICNIRP) and the Institute of Electrical and Electronics Engineers (IEEE) publish guidelines recommending exposure limits to protect the health of the general public and live-line workers against demonstrated effects of electromagnetic fields [21,22]. These guidelines are mostly based on the best available estimate of acute biological effects (e.g., magnetophosphene perception threshold) on humans. Therefore, the primary objective of this pilot study was to evaluate if an acute neurophysiological response could be detected in a highly sensitive neuromotor behavior: physiological tremor. More specifically, the objective was to establish if a 60 Hz MF with high level of magnetic flux density (5–50 mT_rms_) could induce acute modulations in postural tremor. It was hypothesized, based on earlier work [11,23,24], that physiological tremor amplitude would increase in the frequency domain between 8 and 12 Hz (i.e., tremor frequency band associated with the central nervous system contribution) as a consequence of the MF exposure. The secondary objective was to determine which brain region is responsible for the observed changes via recording EEG activity in the primary motor and somatosensory cortices contralateral to the tremor recording. Interestingly, it has already been reported that the EEG alpha rhythm (8–12 Hz), which primarily originates in the occipital cortex [25], can be modulated by ELF MF exposure (see [26,27] for a review). Note that the same 8–12 Hz EEG oscillations are also present in the motor cortex (where it is called the “mu” rhythm), and correlate with motor activity and tactile perception [28,29]. We hypothesized that the EEG alpha activity in the exposed motor cortex would increase with higher levels of MF exposure, and that it would possibly be associated with changes in physiological tremor.

## 2. Materials and Methods

### 2.1. Participants

The experimental protocol was approved by the Health Sciences Research Ethics Board (HSREB approval #103066) of Western University (London, ON, Canada). Ten healthy right-handed individuals (5 Males and 5 Females, age: 23.8 ± 4 SD) were enrolled in the present study. After confirming that all volunteers met the inclusion criteria (18–55 years old, absence of any cardiovascular or neurological disorder or metallic implant, no potential chances of pregnancy, no consumption of street drugs, and refraining from coffee/alcohol/nicotine intake 24 h prior to testing), they had the opportunity to ask any potential questions regarding the experimental procedure. Volunteers had to complete a written consent form in order to participate in the experiment. Then, participants were set up in the experimental environment and were instructed to extend their index finger and hold this position as stable as possible while their contralateral primary motor cortex was exposed to 60 Hz MFs between 5 and 50 mT_rms_ (0–50 mT_rms_ in 5 mT_rms_ increments). Tremor characteristics (amplitude, drift, dominant frequency, median frequency and power in the 8–12 Hz range), as well as EEG mu-rhythm in the C1, C3, C5, CP1, CP3, CP5 electrodes, were measured.

### 2.2. MF Exposure System

**Figure 1:**
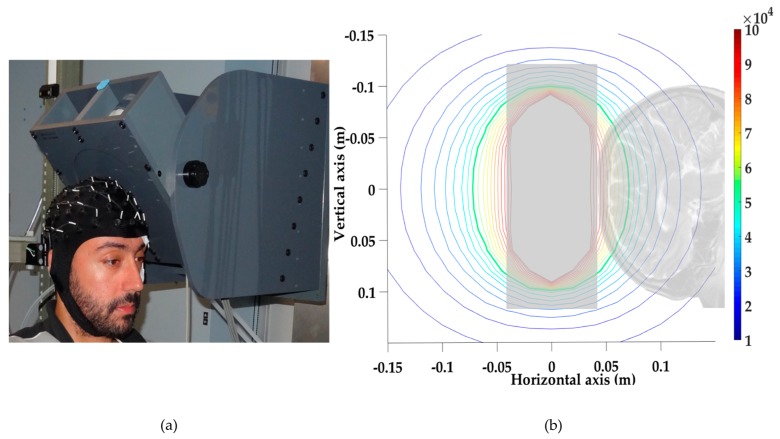
(**a**) Coil (encased in plastic) positioned to expose the motor cortex of a tested participant (centered on the EEG electrode, C3). (**b**) Scaled representation of a sagittal slice of human head superimposed with the field distribution, calculated using the Biot and Savart Law (10% contour lines are 5 mT_rms_ in this case). The vertical transparent grey bar represents the coil dimensions. Brain cortical layers are exposed to a non-homogeneous 60 Hz MF calibrated to deliver 50 mT_rms_ at 3 cm from the surface of the coil (thicker green contour line on the graph, the calculation was confirmed by a direct MF measurement, single axis MF Hall transducer, Senis™ 0YA05F-C.2T2K5J probe, Senis, Baar, Switzerland) ±10 mT_rms_.

A local head exposure system was designed and built at Lawson Health Research Institute (London, ON, Canada). The system was controlled using a custom LabVIEW™ script and a National Instruments A/D Card (National Instruments, Austin, TX, USA), driving a MTS™ Magnetic Resonance Imaging (MRI) gradient amplifier array capable of delivering up to 200 A_rms_ at ±345 V (MTS Automation, Horsham, PA, USA). The local head exposure system consisted of a 176-turn coil (16 layers of 11 turns each, 6 cm inner diameter and 22 cm outer diameter) made of hollow square copper wire cooled by circulating water (see coil illustration in Figure 1a). This coil could generate a 60 Hz MF with a flux density of up to 50 mT_rms_ at 3 cm from the coil face, without any perceptible noise or vibration. The MF distribution produced by the coil was calculated using the Biot and Savart Law, and is presented in Figure 1b. Calculations were confirmed by measurements using a single axis MF Hall transducer (±200 mT_rms_ range with 0.1% accuracy, Senis™ 0YA05F-C.2T2K5J probe, Senis, Baar, Switzerland).

### 2.3. Tremor and EEG Measurement

**Figure 2:**
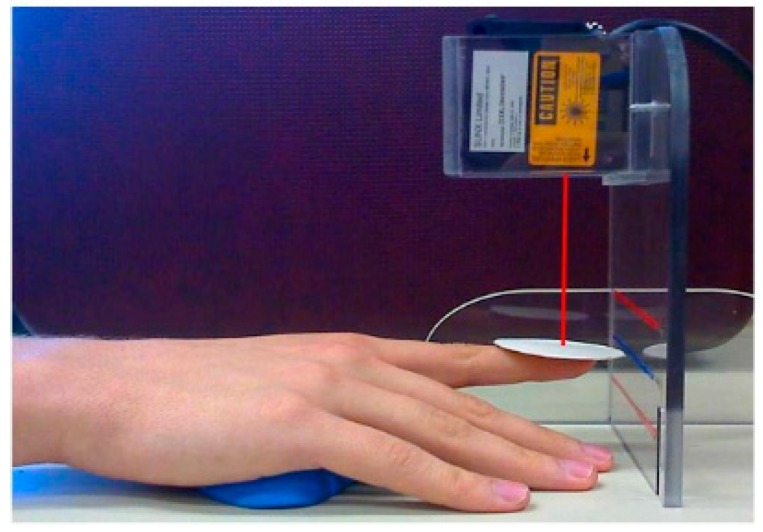
Recording of index finger tremor using the laser sensor. The beam of the laser is pointing down on the middle of the white cardboard while the participant’s palm rests on a piece of molded clay.

Tremor was recorded using a Class II laser diode (Micro laser sensor LM10, series ARN12821, Matsushita Electronic Work Ltd., Osaka, Japan). The acquisition was recorded at a 10 kHz sampling rate via the MRI-compatible EEG amplifier and software (SynAmps2 and SCAN 4.0 respectively, Compumedics-Neuroscan Inc., Charlotte, NC, USA). The laser was placed vertically 8 cm above a round piece of white cardboard (2 cm in diameter and 1.2 g in weight) fixed on the tip of the right index finger and pointing towards the ground to record vertical displacements (Figure 2). The laser resolution was 5 µm after filtering out high frequencies (above 20 Hz-Fast Fourier Transform (fft) and inverse Fast Fourier Transform (ifft)). Five validated tremor characteristics (amplitude, drift, dominant frequency, median frequency and power in the 8–12 Hz frequency range, since this frequency range is indicative of the neurophysiological processes at play) calculated on the recorded time series were converted from volts (V) to millimeters (mm) [12,30,31].

EEG was measured simultaneously with tremor using a 64-channel MRI-compatible EEG cap and a SynAmps2 amplifier (Compumedics-Neuroscan Inc., Charlotte, NC, USA). This technology enabled the recording of EEG signals in the presence of high-level MF exposure. Electrodes were positioned according to the international 10/20 system. EEG data were referenced to a point midway between Cz and CPz, and grounded midway between Fz and FPz. At the start of the recordings impedances were below 20 kΩ. EEG data were sampled at 10 kHz.

This pilot study exclusively focused on the electrical activity of the motor cortex and the primary somatosensory cortex, and therefore only six electrodes were recorded and analyzed: C1, C3, and C5 (covering the left primary motor cortex: M1, controlling the right side of the body), and CP1, CP3, and CP5 (covering the left primary somatosensory cortex: S1, responsible for sensory perception in the right side of the body). The average EEG spectral power in the 8–12 Hz range was calculated for each electrode. Collected raw EEG and tremor time series were imported into MATLAB (The MathWorks Inc., Natick, MA, USA) and were bandpass-filtered (3–25 Hz) using fft and ifft algorithms: a high-pass filter at 3 Hz was followed by a low-pass filter at 25 Hz. The first and last 0.7 s of each exposure interval were discarded to ensure that no edge effects due to the filtering procedure were kept. Prior to final visual inspection, time-series were processed through a standard artifact rejection amplitude-based algorithm (±50 µV at any scalp site, corresponding to usual settings from SCAN software). The final visual examination led to the removal of two recordings from subject 4 (40 mT_rms_ and 45 mT_rms_ both for repetition 5) and one recording from subject 6 (30 mT_rms_, repetition 4). The ‘pwelsh’ function of Matlab was used to compute the power spectral density for each experimental repetition. For each participant, the power spectra resulting from the five repetitions (only four for subjects 4 and 6) of each experimental condition were averaged. The mu-power for each condition was quantified as the integral over the 8–12 Hz frequency band from the averaged spectrum and was used for the statistical analyses.

**Figure 3:**
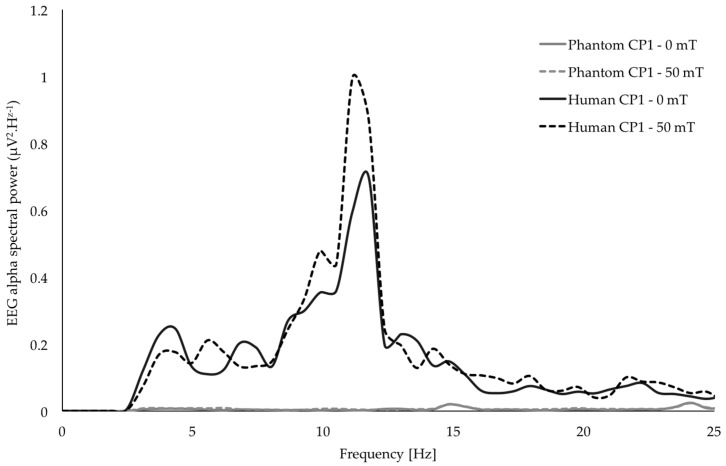
Averaged power spectra for EEG recorded in the CP1 electrode during the 60 Hz exposure, for the 0 and 50 mT_rms_ conditions, in both a phantom and a human (subject 4). Each spectrum is averaged over five repetitions at the corresponding flux density condition. This illustration shows an absence of potential contamination of the EEG in the 3–25 Hz range due to the exposure.

In addition, the entire experimental procedure was replicated on a phantom (watermelon) to confirm that the MRI-compatible hardware and signal post-processing methods were appropriately removing any potential MF-induced artifacts from the EEG data (Figure 3). As illustrated in Figure 3, there was no detectable difference in the EEG spectral content between epochs of MF exposure and sham exposure after processing. Tremor data were free of any artifacts due to the MF exposure system, since the recording system was placed sufficiently far from the coil.

### 2.4. Experimental Protocol

At the beginning of the experimental session participants were equipped with the EEG cap and seated in the experimental armchair. In addition, each participant was provided with earplugs to prevent them from hearing any potential noise produced by the coil during MF exposure. The coil was oriented to expose the motor cortex contralateral to the tested index finger (coil centered on the C3 EEG electrode for all participants, see Figure 1a). EEG and tremor data were collected during each 5-s period of 60 Hz MF exposure delivered between 0 (sham) and 50 mT_rms_ in 5 mT_rms_ increment steps. Each of the eleven exposure conditions was repeated five times, resulting in a total of fifty-five exposure periods of 5 s. Each 5-s exposure period was separated from the next by a 5-s period free of exposure (0 mT_rms_). The order of delivery for the 55 repetitions of exposure periods was randomized and given in a double-blind manner (by the LabVIEW software managing the MF exposure delivery, which was entirely computer-driven). There was no direct interaction between the experimenter and the participant during the entire course of the exposure conditions [30].

During MF exposure epochs, participants performed the physiological postural tremor task. While seated in the experimental armchair, participants rested their dominant arm on the armrest with the palm facing down and positioned on a molded support (Figure 2). Participants were instructed to point their index finger towards a static target displayed on a LCD screen showing instantaneous visual feedback of the finger position. Once the finger position was aligned with the visual target on the screen, room lights were extinguished and participants were instructed to close their eyes for the duration of the test and keep their finger position as steady as possible despite the absence of feedback.

## 3. Results

All statistical results are presented in Table 1. Postural tremor characteristics (amplitude, drift, dominant frequency, median frequency and power in the 8–12 Hz frequency range) and EEG spectral power in the 8–12 Hz frequency range were compared using a one way repeated measures MANOVA (11 variables testing 11 flux density conditions of MF at 60 Hz) using the Greenhouse–Geisser adjustment correcting for non-sphericity (analysis conducted in SPSS Version 24). Post-hoc pairwise comparisons were applied using a Bonferroni confidence interval adjustment for multiple comparisons to variables showing a significant main effect.

**Figure 4:**
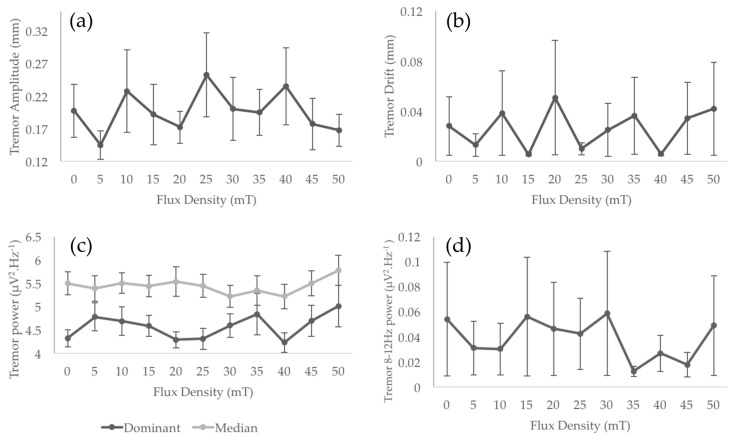
All Tremor characteristics: Tremor amplitude (**a**); tremor drift (**b**); dominant and median frequency spectral power (**c**); and tremor 8–12 Hz spectral power (**d**). None of these characteristics showed significant change with increase in flux density. The four panels represent average values calculated on the ten participants for each MF exposure condition (from 0 to 50 mT_rms_). Error bars at each data point represent standard error of the mean.

**Figure 5:**
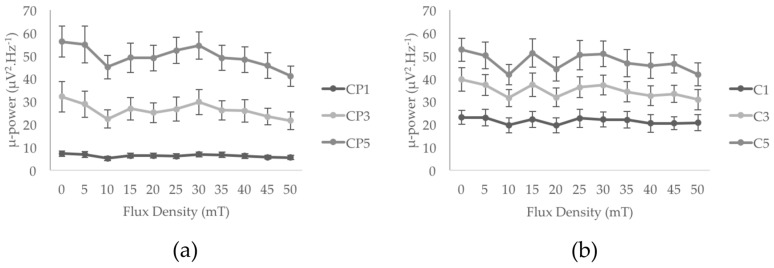
Mu-rhythm (8 to 12 Hz) spectral power: in CP1, CP3, and CP5 (**a**); and in C1, C3 and C5 (**b**) electrodes. CP3 and CP5 electrodes show a significant main MF effect suggesting a decrease of mu-rhythm amplitude associated with increasing flux density (although not confirmed by post-hoc pairwise comparisons). Each data point represents average values calculated on the ten participants for each MF exposure condition (from 0 to 50 mT_rms_). Error bars represent standard error of the mean.

Results did not show any significant main exposure effect for any of the tremor characteristics: amplitude, drift, dominant and median frequencies, 8–12 Hz power (Figure 4 and Table 1). Among the six EEG electrodes of interest, C1, C3, C5, and CP1 did not show any significant main effect of MF exposure (from F = 0.89, *p* > 0.4, ηp^2^ = 0.09, power = 0.24 for C1 to F = 2.49, *p* > 0.05, ηp^2^ = 0.18, power = 0.68 for CP1). However, a significant main effect of MF exposure was found for CP3 (F = 3.48, *p* < 0.05, ηp^2^ = 0.28, power = 0.74) and CP5 (F = 2.94, *p* < 0.05, ηp^2^ = 0.25, power = 0.73). As illustrated in Figure 5a, the increase of flux density appears to be associated with a decrease of the spectral power of the mu-rhythm. It is interesting to notice here that, despite the limited number of participants in this pilot study, these main effects reached statistical powers of, respectively, 0.75 and 0.73, and account for, respectively, 28% and 25% of the variance (see Table 1). Although the data show 33% and 27% decrease of mu-rhythm spectral power between the 0 mT_rms_ and 50 mT_rms_ conditions for CP3 and CP5 electrodes respectively, the Bonferroni confidence interval adjustments of pairwise comparisons did not show any significant differences between the flux density levels for the two electrodes (*p* > 0.1 in all comparisons; see Figure 5).

Acknowledging the pilot character of this work, further sample-size analyses were conducted on both the EEG and tremor characteristics showing the lowest power. According to the observed effect sizes, the mu-rhythm spectral power of the C1 electrode would significantly be affected by the flux density with a statistical power of 0.8 with a total of 30 participants. Regarding the tremor 8–12 Hz spectral power, the same effect would be achieved with a total of 29 participants. These sample size calculations were conducted with G*Power 3.1.9.2. [32].

## 4. Discussion

In previous investigations of the effect of ELF MF on tremor, our group focused on low levels of exposure (1 mT_rms_ at 50 Hz and 1.8 mT_rms_ at 60 Hz) over the whole-body and showed weak or no effect on postural tremor over long periods of time [11,24,30]. It is important to notice that these studies were applying flux densities up to 50 times lower for exposure durations of up to 1 h and the reported effects were occurring after a period of cumulative exposure, as opposed to instantaneously following the presentation of the stimulus in the current work. In addition, the previous tremor characteristics reported as significant are not the same as those calculated in the present paper. For example, in Legros and coworkers’ study [11] the mean significant power was extracted from 2 to 20 Hz tremor time series on a time-frequency analysis using a specific wavelet analysis technique not used here, which cannot be equated to any amplitude value presented in this work. All these differences make it difficult to compare previous results with the results from the current study, where we are looking at acute effects specifically (i.e., short term, immediately after the presentation of the stimulus) of a high-level ELF MF (up to 50 mT_rms_) applied locally to the motor cortex.

In addition, in the current work, EEG recordings of brain activity were simultaneously performed to investigate the possible EEG responses to the stimulus during the tremor task. MRI-compatible hardware (EEG cap and amplifier) synchronizing EEG and tremor recordings made possible this first study of human EEG activity during a 60 Hz MF of up to 50 mT_rms_. The phantom testing confirmed that data were free of MF-induced artifacts in the frequency range of interest for all the characteristics studied, excluding artifact related bias due to the exposure. Overall, and in contrast to our expectations, 60 Hz MF between 0 and 50 mT_rms_ at the level of the sensorimotor/motor cortex did not induce effects on the studied physiological tremor characteristics (amplitude, drift, dominant frequency, median frequency and power in the 8–12 Hz frequency range) in the pilot sample of ten participants. These pilot results suggest that, if there is an effect of the exposure on sensorimotor/motor cortex activity, it does not translate into an associated motor outcome that could be captured through physiological tremor quantification. Interestingly, this is consistent with EEG results showing no effect in the three electrodes located over M1, the region of the motor cortex responsible for motor control in the tested finger. It is important to highlight that the levels of exposure for 60 Hz MF presented in this work are up to 100 times higher than the reference limitation for the general public (200 µT) recommended by ICNIRP [21] and such levels of exposure did not have any acute impact on a sensitive neurophysiological motor outcome such as physiological tremor. Such finding can be accounted as an interesting result in itself.

However, two of the three electrodes located over S1 (involved with tactile perception) show a modulated mu-rhythm with increased MF flux density (CP3 and CP5, and tendency approaching significance for CP1), but this was not confirmed by post-hoc pairwise comparisons. Nevertheless, it is worth noticing that the size of the decrease observed in our study is comparable to the 30% decrease in mu-rhythm reported over the sensorimotor regions between resting state and a hand movement task [33]. The mu-rhythm, also called the sensorimotor rhythm, corresponds to synchronized patterns of electrical activity in the motor cortex predominantly occurring when the body is at rest (i.e., in the absence of movement production or sensory perception). This rhythm can be dampened or even cancelled by performing a motor action or by tactile perception [28,29]. The reduction of the amplitude of this rhythm is attributed to a desynchronization of neurons’ firing as a consequence of an increased solicitation of the motor cortex. A decreased mu-rhythm in the motor cortex region is thus the consequence of increased but desynchronized firing of neural networks in this same region [28,29]. Therefore, it is possible to speculate that high-intensity MF exposure may instantaneously, slightly increase sensory processing in the primary somatosensory cortex. It would however require replicating this pilot work with a sample size of at least 30 human volunteers to achieve a statistical power reaching 80%. Interestingly, similar outcomes have been presented in previous research investigating long exposure of MF at low-intensity, showing increased functional brain activity in this region during a finger-tapping task conducted after thirty minutes and one hour of exposure to a 60 Hz MF at 1.8 and 3 mT_rms_, respectively [34]. Furthermore, a recent study showed that an electrical stimulation between 10 to 14 Hz, and 52 to 70 Hz delivered to the somatosensory cortex using surface electrodes could elicit tactile perceptions in the contralateral hand [35] in the absence of any sensory stimulus. The consistency with our pilot results is interesting, since they also suggest that the somatosensory cortex activity can be modulated by a short high-level MF without mechanical stimulation, yet no tactile sensation was reported and no behavioral effects were exhibited. Nevertheless, even if these results consistently point at a possible modulation of somatosensory cortex rather than the motor cortex itself via MF exposure, the present results must be considered carefully. Due to the small sample size used in this pilot study, this possible somatosensory effect can only be presented as a speculative hypothesis.

## 5. Conclusions

In conclusion, this pilot study suggests a possible effect of 60 Hz MF in the high mT-range (above 10 mT_rms_, up to 50 mT_rms_) on the brain region responsible for tactile perception (somatosensory cortex, S1). Although this effect did not translate into a functional outcome (no physiological tremor modifications), this effect highlights the importance of including a direct measurement of tactile perception in future developments. These pilot results are thus paving the way to proposing documented hypotheses to be tested in a much-needed full study, aiming to provide solid conclusions regarding sub-threshold experimentally defined values under which no effects can be observed. Moreover, this pilot study has only investigated a local 60 Hz exposure, and has not addressed the question of the frequency response (only the 60 Hz frequency was tested here) and of the impact of homogeneous head exposure. As part of a wider research program designed to experimentally establish systematic thresholds for acute human neurophysiological responses, and to systematically explore the underlying mechanisms of action, this pilot study contributes to one general objective: providing new experimental results acquired in humans, which are relevant to international standard/guideline agencies such as ICNIRP or IEEE.

## Figures and Tables

**Table 1:** Statistical results for each variable describing the tremor characteristics and the EEG spectral analysis for electrodes C1, C3, C5, CP1, CP3 and CP5. All results are corrected using the Greenhouse–Geisser adjustments.

Physiological Measure	Characteristic/Channel	F	*p*	Partial Eta Squared	Power
Tremor Characteristics	Amplitude	2.012	0.157	0.183	0.382
	Drift	1.028	0.337	0.103	0.149
	Dominant Frequency	1.215	0.322	0.119	0.255
	Median Frequency	1.359	0.270	0.131	0.364
	8–12Hz Power	0.934	0.360	0.094	0.140
EEG Spectral Power	C1	0.893	0.470	0.090	0.240
	C3	1.994	0.119	0.181	0.528
	C5	2.037	0.107	0.185	0.562
	CP1	2.490	0.053	0.217	0.686
	CP3 (*)	3.485	0.025	0.279	0.748
	CP5 (*)	2.949	0.033	0.247	0.732

* mark variables for which *p* < 0.05.
